# Effect of Wheelchair Frame Material on Users' Mechanical Work and Transmitted Vibration

**DOI:** 10.1155/2014/609369

**Published:** 2014-09-03

**Authors:** Félix Chénier, Rachid Aissaoui

**Affiliations:** ^1^Laboratoire de Recherche en Imagerie et Orthopédie (LIO), Centre de Recherche du Centre Hospitalier de l'Université de Montréal (CRCHUM), Montréal, Canada; ^2^Centre de Recherche Interdisciplinaire en Réadaptation de Montréal, Institut de Réadaptation Gingras-Lindsay, Montréal, Canada; ^3^Département de Génie de la Production Automatisée, École de Technologie Supérieure, Montréal, Canada

## Abstract

Wheelchair propulsion exposes the user to a high risk of shoulder injury and to whole-body vibration that exceeds recommendations of ISO 2631-1:1997. Reducing the mechanical work required to travel a given distance (WN-WPM, weight-normalized work-per-meter) can help reduce the risk of shoulder injury, while reducing the vibration transmissibility (VT) of the wheelchair frame can reduce whole-body vibration. New materials such as titanium and carbon are used in today's wheelchairs and are advertised to improve both parameters, but current knowledge on this matter is limited. In this study, WN-WPM and VT were measured simultaneously and compared between six folding wheelchairs (1 titanium, 1 carbon, and 4 aluminium). Ten able-bodied users propelled the six wheelchairs on three ground surfaces. Although no significant difference of WN-WPM was found between wheelchairs (*P* < 0.1), significant differences of VT were found (*P* < 0.05). The carbon wheelchair had the lowest VT. Contrarily to current belief, the titanium wheelchair VT was similar to aluminium wheelchairs. A negative correlation between VT and WN-WPM was found, which means that reducing VT may be at the expense of increasing WN-WPM. Based on our results, use of carbon in wheelchair construction seems promising to reduce VT without increasing WN-WPM.

## 1. Introduction

Whereas wheelchairs contribute greatly to the physical activity, mobility, and autonomy of their users, wheelchair propulsion is also physiologically detrimental. About half of manual wheelchair users will develop shoulder injuries due to the high mechanical load at the shoulders during propulsion [1–4]. It is believed that improving propulsion efficiency can help in preserving the shoulder function [5–7]. One way to achieve this goal is to propel a more efficient wheelchair, that is, wheelchair that minimizes the required mechanical work to travel a given distance (work-per-meter (WPM)). On the other hand, the regular use of a wheelchair exposes the users to whole-body vibrations and shocks that exceed the recommendations of ISO 2631-1:1997, which may be detrimental for both their comfort and safety [8–10]. This means that, currently, wheelchair design faces a dual challenge in reducing both WPM and vibration transmissibility (VT).

Frame material is believed to have an impact on WPM and VT. Originally, most wheelchairs were made of steel, but alternative materials such as aluminium, titanium, and carbon are now becoming more and more popular, mostly because they allow building lighter and stiffer wheelchairs [11]. However, little is known about the effect of frame material on WPM and VT. The impact of wheelchair weight on WPM is still debated. Lighter wheelchairs are believed to decrease WPM because rolling resistance is proportional to weight. However, de Groot et al. [12] did not observe changes in any kinetic parameter or oxygen uptake when propelling a wheelchair with 5 kg or 10 kg extra. On the other hand, Cowan et al. [13] did not measure power output or WPM but did observe higher peak forces and lower self-selected velocities with 9.1 kg extra. Titanium wheelchairs are believed to transmit less vibration than aluminium wheelchairs [6, 14], but evidence contradicts this claim [15, 16]. Carbon wheelchairs are also thought to transmit less vibration than aluminium wheelchairs, but no study currently exists to prove this claim. Therefore, there is a need for a better understanding of the frame material impact on WPM and VT.

The choice between a folding and a rigid frame also has an impact on WPM and VT, and this choice is really up to the user's preferences. A survey of 549 wheelchair users associated folding wheelchairs with increased shoulder pain compared to rigid wheelchairs when propelling for more than 10 minutes or when propelling up a ramp [7]. In terms of VT, although Garcia-Mendez et al. [9] found no difference between 9 folding wheelchairs and 20 rigid wheelchairs in everyday conditions, Kwarciak et al. [15] found that, for a curb-descending task with 16 wheelchairs, the vibration transmitted by folding wheelchairs was higher than by rigid wheelchairs when acceleration was frequency-weighted as recommended by ISO 2631-1:1997. Therefore, to limit the scope of this work, only folding wheelchairs were considered.

Other wheelchair components also have an impact on VT. Although the impact of rear suspension on VT is mitigated [9, 15, 18–20], caster suspension does reduce lower body vibration [19]. However, as rear suspension is only available on rigid frames and caster suspensions are add-on devices that are not intrinsic to the wheelchair, we excluded suspension from this study. Composite wheels are also very popular devices that are advertised to improve WPM and VT [14]. However, as both claims were not verified [21, 22] and as these wheels are also add-ons that are not intrinsic to the wheelchair, we excluded composite wheels from this study and concentrated only on the effect of frame material.

The purpose of this work is twofold. The first is to measure the effect of frame material on folding wheelchairs' WPM and VT. Following the common belief that carbon and titanium wheelchairs have better vibration properties than aluminium wheelchair [6, 14], we hypothesized that carbon and titanium wheelchairs have lower vibration transmissibility (VT) than aluminium wheelchairs. The second is to investigate a possible compromise between WPM and VT. In fact, if a wheelchair is designed to deform to absorb vibration and shocks, some propulsion energy may be lost in this deformation; thus, reducing VT could increase WPM. We made the second hypothesis that a negative correlation exists between WPM and VT. To verify these hypotheses, six commercially available ultralight folding wheelchairs made of titanium, carbon, and aluminium were compared in terms of both WPM and VT. 10 able-bodied subjects propelled the six wheelchairs on three ground surfaces. WPM was measured using two instrumented rear wheels, and VT was measured using five accelerometers placed into the seat cushion and on the frame.

## 2. Methodology

ISO 2631-1:1997 standard on mechanical vibration and shock [8] defines the methods to assess the user's vibration exposure in seated position, which is defined by these parameters.

*a*_*w*_ : root-mean-square (RMS) value of the frequency-weighted acceleration (base analysis): this figure is related to the continuous vibration transmitted to the user and is expressed in m/s^2^.
VDV:
vibration dose value (complementary analysis): this figure is related to the shock-induced vibration transmitted to the user and is expressed in m/s^1.75^.

Principal requirements of the norm are as follows.

1. Measurements are made in situations approaching real-life conditions.
2. Measurements are made at the interface between the user and the source of vibration.
3. Measurements are made orthogonally to the ground.
4. Measurements bandwidth must cover the 1 to 80 Hz spectrum.

This norm was followed at different extents in wheelchair propulsion studies [9, 10, 23]. A variant of this norm was also used in DiGiovine et al. [24] and Garcia-Mendez et al. [25] to measure the vibration transmissibility of seat cushions. They made the ratio between the vibration measured on the subject (output) and the vibration measured below the seat (input). In our case, this can be adapted to measure the wheelchair frame VT, by comparing the vibration measured at the seat-user interface (output) to the vibration measured at the four wheels hubs (inputs).

It is important to note that ISO 2631-1:1997 is based on studies performed with able-bodied persons, subjected to vibration in a passive way. As such, it cannot be completely generalized to wheelchair propulsion, and it should be interpreted carefully when used on the SCI population. A discussion on the application of ISO 2631-1:1997 to wheelchair propulsion is given in the appendices.

ASTM F1951 standard specification for determination of accessibility of surface systems under and around playground equipment [26] uses the work-per-meter (WPM) method described in Chesney and Axelson [17] to compare the mechanical work required to propel the same wheelchair on different ground surfaces, based on instrumented wheels data. The wheelchair must be propelled from stop on a 2 m surface and stop by itself exactly at the end of the two meters. This requirement is to ensure that all the energy produced as work by the user is exhausted at the end of the run. This however requires propelling at a very low average speed of 0.3 m/s and thus violates the first requisite of ISO 2631-1:1997 that measurements must be representative of real-life conditions.

A similar method was used in Cooper et al. [23] to measure the average mechanical work required to traverse different surfaces, but at a steady state average velocity of 1 m/s over a 7.6 m long surface. However, they did not specify if the final velocity was always equal to the initial velocity, which is very important. In fact, from conservation of energy, we have

(1) $$\begin{array}{c} E_{0}+W_{\text{nc}}=E_{1}, \end{array}$$

where *E*_0_ and *E*_1_ are the initial and final total mechanical energy and *W*_nc_ is the work done by nonconservative forces. *W*_nc_ can be expressed as

(2) $$\begin{array}{c} W_{\text{nc}}=W_{\text{prop}}+W_{\text{res}}, \end{array}$$

where *W*_prop_ is the propulsion work generated at the wheels by the user and *W*_res_ is the resistive work dissipated by friction and vibration. To measure *W*_res_ using instrumented wheels, one must be sure that *E*_1_ = *E*_0_, so that |*W*_res_ | = |*W*_prop_|. Propulsion work is obtained by

(3) $$\begin{array}{c} W_{\text{prop}}=\overset{\theta_{R1}}{\underset{\theta_{R0}}{\int }}M_{R}d\theta_{R}+\overset{\theta_{L1}}{\underset{\theta_{L0}}{\int }}M_{L}d\theta_{L}, \end{array}$$

where *M*_*R*_ and *M*_*L*_ are the right and left moments applied on the wheels by the user, *dθ*_*R*_ and *dθ*_*L*_ are the right and left wheel angle variations, and *θ*_*R*0_, *θ*_*R*1_, *θ*_*L*0_, and *θ*_*L*1_ are the initial and final angular position of right and left rear wheels. WPM is obtained by dividing *W*_prop_ by the travelled distance *L*.

In this work, WPM was calculated using a realistic steady state average velocity of 1 m/s on a 7 m run. After each recording, we kept only a 5 m interval within these 7 m where the final velocity was equal to the initial velocity. Contrarily to the ASTM F1951 standard, multiple users performed the same task. Therefore, to take the differences of body and wheelchair weights into account, WPM was weight-normalized by the total mass of the users and wheelchairs, so that

(4) $$\begin{array}{c} \text{WN-WPM}=\frac{\int_{\theta_{R0}}^{\theta_{R1}}M_{R}d\theta_{R}+\int_{\theta_{L0}}^{\theta_{L1}}M_{L}d\theta_{L}}{Lm}, \end{array}$$

where WN-WPM stands for weight-normalized work-per-meter, *L* is the distance (5 m), and *m* is the mass.

### 2.1. Material

A total of six folding wheelchairs were tested and compared. To measure the impact of frame material on WN-WPM and VT, three similar wheelchairs featuring a single cross-brace folding mechanism were compared: one made of titanium (Ti), one made of carbon (C), and one made of aluminium (Al1). To consider the effect of folding design on WN-WPM and VT, three additional aluminium wheelchairs (Al2, Al3, and Al4) were tested, each featuring a different folding mechanism.  Table 1 lists these wheelchairs along with their weight, material, and folding design.

**Figure 1:**
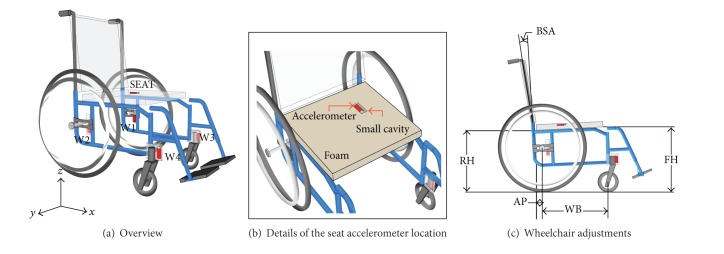
Accelerometers placement on the wheelchairs.

All wheelchairs used the same wheels. Solid tires were used on all wheels, so that tire pressure did not need to be monitored. Wheelchairs were weighed without rear wheels and seat cushion using an AMTI-OR6 force platform with a resolution of 150 g. Weight distribution on the wheels was not verified using a force platform, but wheelchairs were adjusted equally according to Figure 1(c), with an anteroposterior seat position (AP) of 4.4 ± 0.3 cm, a rear seat height (RH) of 41.0 ± 0.6 cm, a front seat height (FH) of 44.9 ± 2.0 cm, a wheel base (WB) of 48.4 ± 2.8 cm, a wheelchair width (including handrims) of 66.2 ± 4.0 cm, a rear wheels diameter of 60 cm, and a backseat angle (BSA) of 1.7 ± 2.6 degrees. Although backrest supports were different from one wheelchair to another (the stock backrest supports were used), the same midrange foam seat cushion with a width of 3 cm was used on every wheelchair.

Five triaxial piezoelectric accelerometers with a bandwidth of 240 Hz (VR001, Midé Technology) were installed on the wheelchairs as shown in Figure 1 and were sampled at 3200 Hz. Accelerometers W1 to W4 were installed on small aluminium plates fixed on the frame, so that they recorded the vibration induced on the frame by the wheels. Accelerometer SEAT was installed into a small cavity above the seat cushion, just below the user's left ischion. This accelerometer recorded the vibration transmitted to the user. A SIT-BAR indenter was not used because it would have modified the pressure distribution at the seat-user interface, which is not allowed by ISO 2631-1:1997. All accelerometers were installed orthogonally to the ground and wheelchair.

Rear wheels were instrumented wheels (SmartWheel, Out-Front Corp.), weighing 4.73 kg each. Forces and moments applied by the user on the rear wheels were sampled at 240 Hz, along with the angular position of the rear wheels.

### 2.2. Methods of Experiment

The experimental protocol was approved by the Research Ethics Committee (CÉR) of the École de Technologie Supérieure (ÉTS). 10 able-bodied subjects were recruited for this experiment, which took place at ÉTS. Subjects distribution was 6 men and 4 women, with an average weight of 73.4 ± 13.3 kg, an average height of 173.9 ± 8.1 cm, and an average body-mass index (BMI) of 24.2 ± 3.3 kg/m^2^. After giving their informed consent, the subjects were instructed to practise propelling a random wheelchair at 1 m/s. This velocity was first controlled with a chronometer, and subjects were then asked to try to keep this velocity during all trials.

After the period of familiarization, subjects were asked to sit on one of the six instrumented wheelchairs and to propel successively on three different ground surfaces:

1. smooth vinyl floor;
2. textured rubber mat with a diamond-shaped pattern (1 mm thick diamonds, 2200 diamonds per square meter);
3. obstacle: smooth vinyl floor with one bump (rectangular section, 5 mm thick, 30 mm wide).

**Figure 2:**
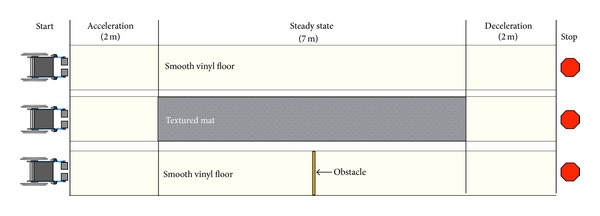
Testing conditions.

Testing conditions are shown in Figure 2. For each condition, subjects started 2 meters away from the surface, accelerated until they reached their steady state velocity, and propelled on the 7 meters of the surface. Each trial was performed three times. After propelling on each surface, data were transferred to a computer and the accelerometers and instrumented wheels were installed on the next wheelchair. Subjects were then asked to repeat the same steps with the new wheelchair.

Every subject propelled all six wheelchairs on the three ground surfaces. Wheelchairs and ground surfaces order were randomized to avoid a bias due to the fatigue of the subjects. At the end, a total of 540 trials were analyzed (10 subjects × 6 wheelchairs × 3 surfaces × 3 trials).

### 2.3. Processing of Vibration Data

All data processing was performed with Matlab (The Mathworks, Inc., Natick, MA). For each trial, the following steady state data were kept for analysis.

*Smooth Vinyl Floor*. From the start of the third push to the end of the last push. 

*Textured Rubber Mat*. From the start of the third push, as long as the wheelchair was on the textured mat.

*Smooth Vinyl Floor with Bump*. Two seconds before and after the front wheels roll over the bump.

Parameters *a*_*w*_ and VDV were then calculated on the three axes of each accelerometer:

(5) awi(ACC)=(1T∫0T(wi(t)∗ai(ACC)(t))2dt)1/2,VDVi(ACC)=(∫0T(wi(t)∗ai(ACC)(t))4dt)1/4,

where *i* is the axis (*x*, *y*, *z*), ACC is the accelerometer identifier (SEAT, W1, W2, W3, or W4), *a*_*i*(ACC)_(*t*) is the acceleration measured on axis *i* in m/s^2^, *w*_*i*_(*t*) is the impulse response of the frequency weighting transfer functions given by ISO 2631-1:1997, *T* is the total recording time, and ∗ is the convolution operator.

ISO 2631-1:1997 states that vibration should be reported on the axis with the highest vibration. However, a special case is also accepted when vibration is comparable on two axes, in which case both vibration values can be combined into a total vibration. In our data, the average vibration at the seat for every trial was highest on the *z*-axis but comparable to the *x*-axis. Vibration on the *y*-axis was about 80% lower than *x* and *z*. Literature does not agree on the choice between the vertical (*z*) vibration or the total (*x*-*z*) vibration for wheelchair vibration assessment [9, 10, 23, 24]. Therefore, both were calculated. The choice between one or the other is discussed in the appendices. Total (*x*-*z*) vibration was calculated as follows:

(6) awt(ACC)=kx2awx(ACC)2+kz2awz(ACC),VDVt(ACC)=kx4VDVx(ACC)4+kz4VDVz(ACC)44,

where *k*_*x*_ = 1.4 and *k*_*z*_ = 1 [8].

In total, eight vibration parameters were assessed. The vibration transmitted to the user by the seat was defined by the following four parameters:

 
*a*_*wz*(SEAT)_: vertical (*z*) continuous vibration (m/s^2^),
 VDV_*z*(SEAT)_: vertical (*z*) shock-induced vibration (m/s^1.75^),
 
*a*_*wt*(SEAT)_: total (*x*-*z*) continuous vibration (m/s^2^),
 VDV_*t*(SEAT)_: total (*x*-*z*) shock-induced vibration (m/s^1.75^).

The vibration transmissibility (VT) of the frame (%) was defined by the following four parameters:

 VT_*awz*_: vertical (*z*) continuous vibration transmissibility,
 VT_VDV_*z*__: vertical (*z*) shock-induced vibration transmissibility,
 VT_*awt*_: total (*x*-*z*) continuous vibration transmissibility,
 VT_VDV_*t*__: total (*x*-*z*) shock-induced vibration transmissibility,

where

(7) VTaw{z,t}=aw{z,t}(SEAT)(1/4)∑i=14aw{z,t}(Wi(×100%,VTVDV{z,t}=VDV{z,t}(SEAT)1/4∑i=14VDV{z,t}(Wi)×100%.

For each subject, wheelchair, and surface, the mean of each parameter was taken over the three trials.

### 2.4. Processing of Mechanical Work Data

The mechanical work was assessed on the smooth vinyl floor and on the textured rubber mat. Mechanical work analysis used the same steady state data as vibration analysis. For each trial, we kept a 5 m subset where the final wheelchair velocity was equal to the initial wheelchair velocity. Weight-normalized work-per-meter (WN-WPM) was then computed from (4). For each subject, wheelchair, and surface, the mean of the WN-WPM was taken over the three trials.

### 2.5. Statistical Analysis

Lilliefors tests were performed to assess the normality of WN-WPM values and every 8 vibration parameters values. Tests were performed independently for the six wheelchairs. We found that normality cannot be rejected (*P* < 0.05) for the majority of our samples; therefore, a parametric statistical analysis was selected. For each parameter, an analysis of variance (one-way ANOVA) was performed over the six wheelchairs. When the ANOVA resulted in a *P* value below 0.05, a Tukey-Kramer post hoc test was performed to determine which wheelchair(s) stood out from the others. Statistical analysis was performed under Matlab using  anova1  and  multcompare methods.

## 3. Results

### 3.1. Vibration

**Figure 3:**
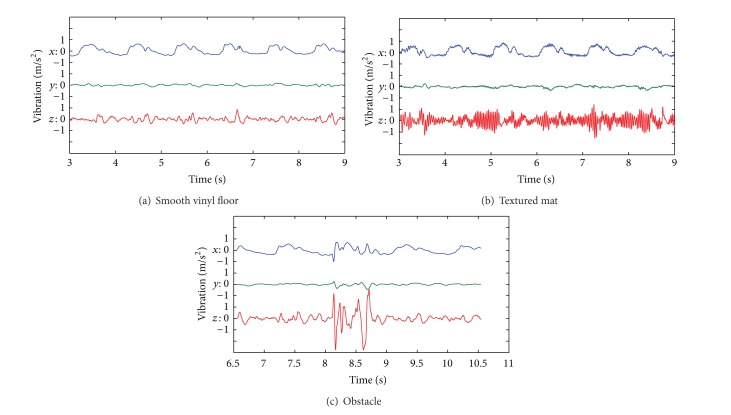
Samples of the triaxial steady state frequency-weighted acceleration recorded at the seat for the three surfaces.

Figure 3 shows samples of the steady state frequency-weighted vibration recorded on the three axes for the three surfaces. The shape of the signals was similar for all subjects and wheelchairs. On these samples, the main source of anteroposterior (*x*) vibration appears to be the self-induced acceleration and deceleration of the wheelchair due to propulsion. We also observe that mediolateral (*y*) vibration is negligible compared to the other axes. Finally, the main effect of vibration due to the textured mat or obstacle appears to be on the vertical axis (*z*).

Table 2 shows the average values for the crest factor, MTVV_*z*_/*a*_*wz*_, and VDV_*z*_/*a*_*wz*_ *T* ^1/4^, which are defined in ISO 2631-1:1997. If, for a given condition, these values are equal to or greater than 9, 1.5, and 1.75, respectively, a complementary vibration analysis is required (VDV). Table 2 shows that this complementary analysis is indeed justified.

Table 3 shows the average vibration transmitted to the subjects when rolling on the three surfaces. No significant difference between wheelchairs was observed in any situation.

Table 4 shows the average vibration transmissibility (VT) of each wheelchair when rolling on the three surfaces. Contrarily to Table 3, significant differences between wheelchairs were observed. In terms of continuous vibrations (*T*_*aw*_),

1. on the smooth vinyl floor, C and Al1 had a lower vertical (*z*) VT than Ti and Al3;
2. on the textured mat, C, Al1, Al2, and Al4 had a lower vertical (*z*) VT than Al3;
3. on the textured mat, C, Al2, and Al4 had a lower total (*x*-*z*) VT than Al3;
4. on the obstacle, a significant difference of total (*x*-*z*) continuous VT was observed between wheelchairs; however, the post hoc test did not allow discriminating one wheelchair from the others.

In terms of shock-induced vibrations (*T*_VDV_),

1. on the smooth vinyl floor, C and Al1 had a lower vertical (*z*) VT than Ti and Al3;
2. on the smooth vinyl floor, Al2 had a lower vertical (*z*) VT than Al3;
3. on the textured mat, all wheelchairs had a lower vertical (*z*) VT than Al3;
4. on the textured mat, C, Al2, and Al4 had a lower total (*x*-*z*) VT than Al3;
5. on the obstacle, a significant difference of total (*x*-*z*) continuous VT was observed between wheelchairs; however, the post hoc test did not allow discriminating one wheelchair from the others.

### 3.2. Mechanical Work

Table 5 shows the average WN-WPM values for all wheelchairs on the smooth floor and on the textured mat. No significant difference was observed between wheelchairs (*P* > 0.1 for the smooth floor and 0.05 < *P* < 0.01 for the textured mat).

### 3.3. Relation between WN-WPM and VT

**Figure 4:**
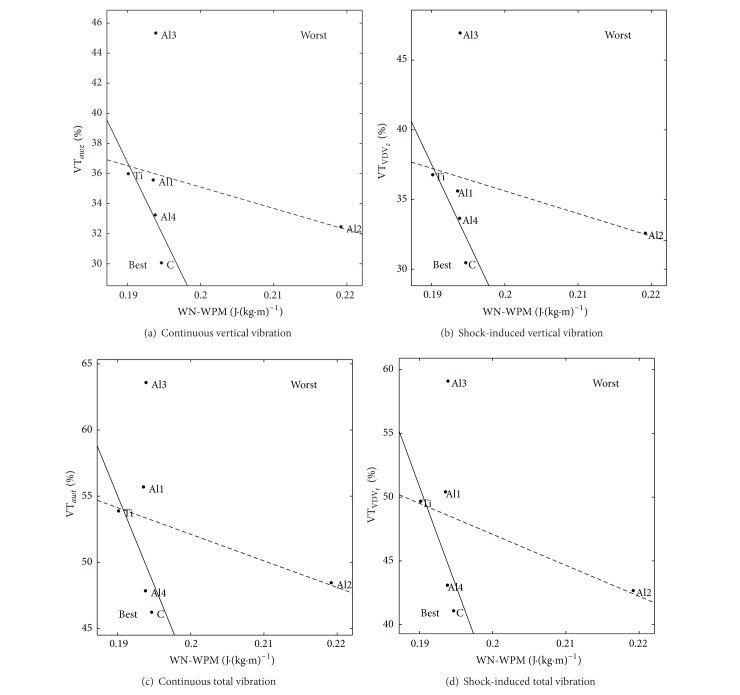
Vibration transmissibility (VT) versus weight-normalized work-per-meter (WN-WPM) when propelling on a textured mat.

Figure 4 shows the average of all trials for each wheelchair, where the *x*-axis is the weight-normalized work-per-meter and the *y*-axis is the vibration transmissibility. A wheelchair situated at the left requires less mechanical work to travel the same distance, and a wheelchair situated at the bottom has lower vibration transmissibility. Therefore, the best wheelchair in terms of both parameters is the nearest to the origin. We selected the propulsion on the textured mat instead of smooth vinyl, because this combination maximized the differences between wheelchairs on both VT and WN-WPM.

A first-order regression between VT and WN-WPM is shown as a dashed line in Figure 4. A small negative correlation was observed between VT and WN-WPM, with a coefficient of correlation ranging from −0.29 to −0.42 in the four cases of Figure 4. We found that the group composed of Al2 and Al3 stood out compared to Ti, C, Al1, and Al4 and thus may be considered as outliers. When Al2 and Al3 were removed, the coefficient of correlation increased to a range of −0.68 to −0.83. The first-order regression with Al2 and Al3 removed is shown as a solid line in Figure 4. These results suggest that VT is indeed negatively correlated to WN-WPM.

## 4. Discussion

### 4.1. Vibration

In Table 3, we observed that, for all parameters, the vibration was higher on the textured mat and on the obstacle compared to the smooth vinyl floor. This was expected as the smooth vinyl floor was not expected to provide significant vibration or shocks. This observation also matches samples in Figure 3. Although vibration at the seat differed between surfaces, we observed no significant differences between wheelchairs in any of the four vibration parameters shown in Table 3. This can be explained partly by the overall good quality of the chosen wheelchairs and by the variation of velocity between trials (see Section 4.4).

We however observed significant differences of vibration transmissibility between wheelchairs (Table 4). In these cases, we believe that the variability of the measurements at the seat was compensated by the same variability at the inputs. It is however impossible to tell how much VT has a real impact on the health risk of the user.

We also observed in Table 4 that VT was often higher than 100% on the smooth vinyl floor, which means that measured vibration at the seat was greater than input vibrations. We believe that, in this particular case with low induced vibration, the seat compression and decompression due to wheelchair propulsion, which is itself a low-frequency vibration, may be nonnegligible compared to input vibrations.

**Figure 5:**
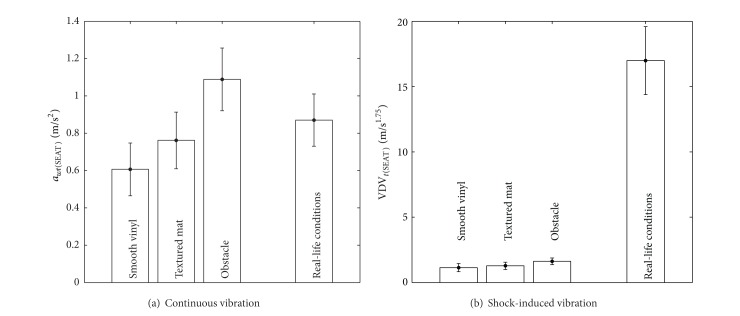
Comparing our total (*x*-*z*) vibration measurements to on-the-field data from Garcia-Mendez et al. [9].

Figure 5 compares the average total (*x*-*z*) vibration between the three tested surfaces and the real-life conditions measured by Garcia-Mendez et al. [9]. Continuous vibration is similar to real-life conditions, but shock-induced vibration is much lower in our study. One explication is that shock-induced vibration (vibration dose value (VDV)) is a cumulative measure that always grows during a measurement period [27]. Therefore, as their measurement period was an entire day while ours was 4 seconds, this explains why they measured higher VDVs. Additionally, the placement of the accelerometer was different between both studies. Garcia-Mendez et al. [9] placed the accelerometer below the seat; therefore, the vibration absorption by the seat was not taken into account in their measure.

**Figure 6:**
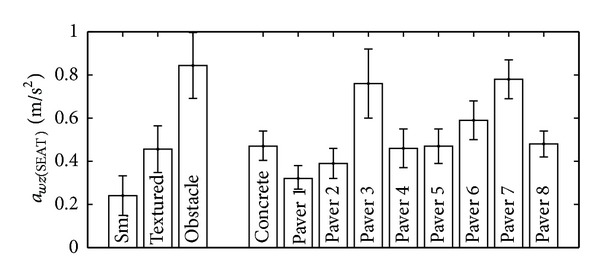
Comparing our vertical continuous vibration measurements to concrete/brick surfaces from Wolf et al. [10].

Figure 6 compares the vertical (*z*) continuous vibration between the three tested surfaces and nine concrete and brick surfaces tested by Wolf et al. [10]. Shock-induced vibration values were not presented in Wolf et al. [10], but continuous vibration values are comparable between both studies. As for Garcia-Mendez et al. [9], Wolf et al. [10] placed their accelerometer below the seat while we placed ours above the seat. Therefore, our data should normally be slightly lower than theirs due to the vibration absorption of the seat. This is however impossible to verify because traversed surfaces were different.

### 4.2. Mechanical Work

A high *P* value was obtained for both surfaces. This means that whereas Ti minimized the mechanical work (0.188 J·(kg·m)^−1^ on the textured surface) compared to Al2 (0.219 J·(kg·m)^−1^ on the textured surface), such differences are statistically nonsignificant and strong conclusions on WN-WPM could not be drawn.

**Figure 7:**
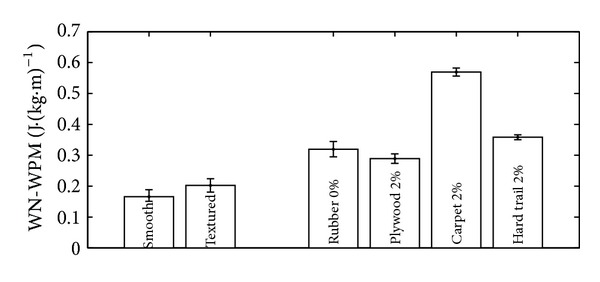
Comparing our weight-normalized work-per-meter measurements to selected surfaces from Chesney and Axelson [17]. Percentage indicates the ascending ramp grade.

The mechanical work to traverse different surfaces was measured by Cooper et al. [23], but as the total distance was not specified, their data cannot be compared with ours. Chesney and Axelson [17] also measured WPM for different surfaces. Some of these surfaces are compared with ours in Figure 7. We observe that the work was always higher in their study; however, most of their comparable surfaces were on a 2% grade ramp, which necessarily needs more work to traverse because part of the work generated by the user is stored as potential energy gains.

### 4.3. Relation between WN-WPM and VT

In Figure 4, we observed that Al2 and Al3 stood out both in terms of WN-WPM and VT from the other wheelchairs. As all wheelchairs were equally configured, their different behaviour could be due to a combination of weight difference (they were the two heaviest tested wheelchairs) or due to frame design difference. When these wheelchairs were removed, the correlation between VT and WN-WPM rose to a range of −0.68 to −0.83. This quite high negative correlation supports our hypothesis that a wheelchair that transmits less vibration requires more mechanical work to traverse the same distance. This result could not be compared with actual literature because vibration and mechanical work were never assessed simultaneously and compared between wheelchairs before.

The titanium wheelchair (Ti) was not found to absorb vibration better than aluminium wheelchairs, which contradicts our hypothesis and current belief but concords with Kwarciak et al. [15] and Cochran [16]. However, it was the wheelchair that demanded the least mechanical work. As only one titanium wheelchair was tested, more wheelchairs should be tested before generalizing this observation to titanium wheelchairs in general.

This is the first time a wheelchair made of carbon fibre was tested for its vibration transmissibility. The carbon wheelchair (C) had the lowest VT, and there was no added work related to this improvement. A comparison between C and Al1, which feature very similar geometry, supports the role of frame material in this lower vibration transmissibility. Whereas only one carbon wheelchair was tested, this encouraging result means that carbon wheelchairs should be studied more thoroughly for the aspects of vibration transmissibility and mechanical work.

We observed in all cases of Figure 4 that the span of Al1, Al2, Al3, and Al4 (same material, different folding design) over WN-WPM and VT was always at least equal to the span of Ti, C, and Al1 (same folding design, different materials). As all wheelchairs were similar in dimensions, adjustments, and weight, this suggests that the folding design may be as important as the frame material when optimizing mechanical work and vibration transmissibility. Additional research on different frame design should be envisaged to confirm this observation.

### 4.4. Study Limitations

The following limitations were identified in this study.

#### 4.4.1. Speed Control

Although wheeling velocity was controlled at 1 m/s during the familiarization, it was not controlled subsequently. Therefore, it varied slightly between trials, with an overall average and standard deviation of 1.00 ± 0.31 m/s. As wheeling velocity does have an effect on vibration [28], this variation may have altered the reproducibility between trials. Wolf et al. [29] controlled time to complete a trial at ±0.5%, while others did not control velocities at all [24, 25, 30]. For future work, we believe that velocity should be controlled during all trials.

#### 4.4.2. Placement of the Seat Accelerometer

We initially chose to place the seat accelerometer in a cavity above the seat so that it was directly coupled with the user. We believe this is the best placement to measure the vibration transmitted to the user. However, when the outcome measure is the frame transmissibility, it would be more advisable to place the accelerometer below the seat, as did Garcia-Mendez et al. [9] and Wolf et al. [10], so that seat cushion absorption is not measured. For future work on vibration transmission, we advise placing an additional accelerometer below the seat. By using two seat accelerometers (one above, one below), it will be possible to isolate the frame and seat vibration transmissibility.

#### 4.4.3. Mechanical Work

The mechanical work was measured only in steady state. Whereas the start-up work (mechanical work required to initiate the movement) was not measured, we believe this value would be of great interest. In fact, higher propulsion moments are required on start-ups than on steady state because of the additional inertial forces caused by the weight of the subject and wheelchair. Therefore, lighter wheelchairs may require less work to initiate movement than heavier ones.

#### 4.4.4. Population

This work was done with able-bodied subjects who had not driven a wheelchair before. As the centre of mass and wheeling technique differ between wheelchair users and nonusers, future studies should also be done with wheelchair users.

## 5. Conclusion

We compared the vibration transmissibility (VT) and the weight-normalized work-per-meter (WN-WPM) of six folding wheelchairs propelled by ten able-bodied users on three ground surfaces. We found significant differences in VT (*P* < 0.05) between wheelchairs, but not in WN-WPM (*P* < 0.1). With both parameters considered at the same time, Ti, Al1, Al4, and C performed better than Al2 and Al3. A negative correlation between vibration transmissibility and mechanical work was found, which supports our hypothesis that a wheelchair that transmits less vibration requires more mechanical work. More wheelchairs should be tested to confirm this correlation. Based on our results, use of carbon in wheelchair construction seems promising to reduce VT without increasing WN-WPM. On the other hand, the titanium wheelchair did not have a lower VT than aluminium wheelchairs, which is in contrast with current belief. As only one carbon wheelchair and one titanium wheelchair were tested, more research on wheelchairs made of these materials should be considered to confirm these observations. For future studies, we recommend giving special attention to wheeling velocity control, placing accelerometers above and below the seat to isolate frame and seat vibration transmissibility, and including start-up work as an additional analysis of mechanical work.

## Figures and Tables

**Table 1:** Properties of the tested wheelchairs.

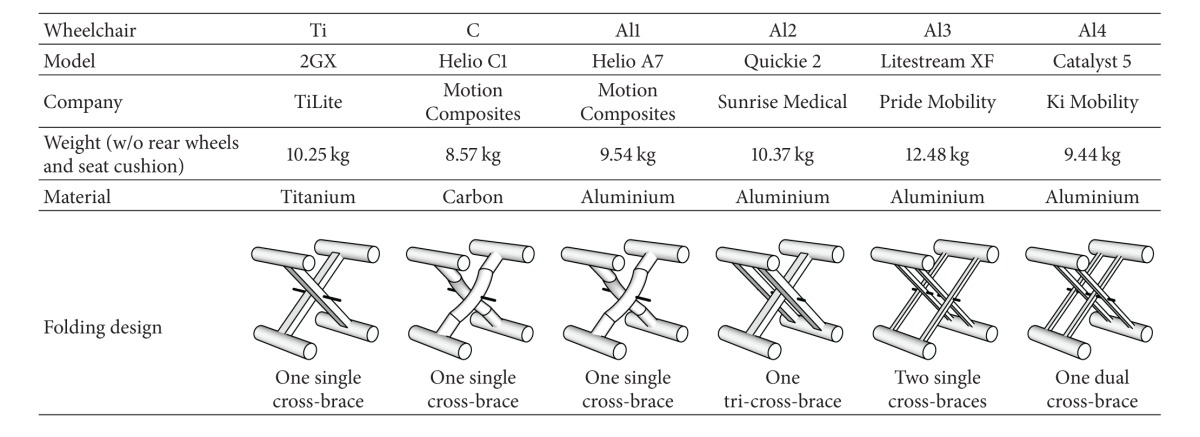

**Table 2:** Requirements for complementary analysis.

	Smooth vinyl floor	Textured mat	Obstacle
Crest factor on vertical axis (max. 9)	5.14	3.38	6.10
MTVV_*z*_/*a*_*wz*_ (max. 1.5)	**1.55**	**1.48**	**1.48**
VDV_*z*_/*a*_*wz*_ *T* ^1/4^ (max. 1.75)	1.49	1.33	**2.01**

Bold values indicate that a complementary analysis (VDV calculation) is required.

**Table 3:** Vibration transferred to the user.

WC	*a*_*wz*(SEAT)_	VDV_*z*_	*a*_*wt*(SEAT)_	VDV_*t*_
	(m/s^2^)	(m/s^1.75^)	(m/s^2^)	(m/s^1.75^)
Smooth vinyl floor				
Ti	0.25 ± 0.07	0.56 ± 0.14	0.63 ± 0.13	1.19 ± 0.31
C	0.25 ± 0.11	0.58 ± 0.27	0.63 ± 0.12	1.15 ± 0.26
Al1	0.22 ± 0.09	0.54 ± 0.21	0.60 ± 0.13	1.15 ± 0.30
Al2	0.25 ± 0.08	0.58 ± 0.18	0.61 ± 0.13	1.15 ± 0.29
Al3	0.24 ± 0.07	0.61 ± 0.21	0.57 ± 0.16	1.09 ± 0.38
Al4	0.23 ± 0.12	0.53 ± 0.25	0.60 ± 0.16	1.12 ± 0.29
Av.	** 0.24 ± 0.09 **	** 0.57 ± 0.22 **	** 0.61 ± 0.14 **	** 1.14 ± 0.31 **

Textured rubber mat				
Ti	0.44 ± 0.08	0.92 ± 0.17	0.76 ± 0.17	1.28 ± 0.30
C	0.44 ± 0.10	0.91 ± 0.20	0.74 ± 0.12	1.24 ± 0.24
Al1	0.40 ± 0.11	0.84 ± 0.21	0.72 ± 0.12	1.21 ± 0.23
Al2	0.48 ± 0.11	0.98 ± 0.19	0.79 ± 0.13	1.31 ± 0.21
Al3	0.46 ± 0.10	1.00 ± 0.22	0.77 ± 0.19	1.31 ± 0.34
Al4	0.50 ± 0.12	1.01 ± 0.24	0.79 ± 0.14	1.32 ± 0.26
Av.	** 0.46 ± 0.11 **	** 0.94 ± 0.22 **	** 0.76 ± 0.15 **	** 1.28 ± 0.27 **

Obstacle				
Ti	0.78 ± 0.11	1.43 ± 0.22	1.02 ± 0.11	1.55 ± 0.18
C	0.86 ± 0.13	1.50 ± 0.15	1.11 ± 0.19	1.60 ± 0.17
Al1	0.79 ± 0.17	1.41 ± 0.26	1.04 ± 0.16	1.52 ± 0.23
Al2	0.79 ± 0.10	1.52 ± 0.21	1.04 ± 0.15	1.61 ± 0.21
Al3	0.87 ± 0.16	1.57 ± 0.35	1.12 ± 0.20	1.66 ± 0.34
Al4	0.86 ± 0.16	1.56 ± 0.35	1.08 ± 0.15	1.64 ± 0.32
Av.	** 0.83 ± 0.15 **	** 1.50 ± 0.28 **	** 1.07 ± 0.17 **	** 1.60 ± 0.26 **

**Table 4:** Vibration transmissibility of the frame (%).

WC	*T*_*awz*_	*T*_VDV_*z*__	*T*_*awt*_	*T*_VDV_*t*__
Smooth vinyl floor				
Ti	172.7 ± 9.0	163.9 ± 13.0	103.3 ± 11.5	99.3 ± 10.7
C	127.9 ± 26.7	126.4 ± 35.0	104.8 ± 8.3	101.4 ± 9.0
Al1	121.7 ± 18.2	115.3 ± 16.7	103.6 ± 8.3	100.3 ± 10.4
Al2	143.7 ± 15.4	138.7 ± 16.1	100.4 ± 8.8	96.9 ± 10.4
Al3	171.2 ± 26.7	175.2 ± 35.2	105.3 ± 11.0	101.7 ± 11.1
Al4	148.8 ± 19.8	140.8 ± 24.0	101.3 ± 10.8	98.0 ± 11.5
Av.	146.4 ± 27.9**	142.1 ± 32.3**	103.1 ± 9.9	99.6 ± 10.7

Textured rubber mat				
Ti	36.0 ± 5.4	36.8 ± 5.7	53.9 ± 6.9	49.7 ± 7.9
C	30.1 ± 6.1	30.5 ± 6.2	46.2 ± 7.6	41.1 ± 8.0
Al1	35.6 ± 7.3	35.6 ± 6.9	55.7 ± 5.8	50.4 ± 6.7
Al2	32.5 ± 5.7	32.6 ± 5.2	48.5 ± 5.2	42.7 ± 5.2
Al3	45.3 ± 8.0	46.9 ± 9.8	63.6 ± 11.3	59.1 ± 13.6
Al4	33.2 ± 7.3	33.7 ± 7.1	47.8 ± 5.6	43.1 ± 5.3
Av.	35.5 ± 8.3**	36.1 ± 8.8**	52.6 ± 9.6**	47.7 ± 10.4**

Obstacle				
Ti	60.3 ± 9.9	47.1 ± 8.8	68.3 ± 7.3	49.8 ± 7.4
C	54.7 ± 5.7	42.4 ± 6.2	62.2 ± 4.1	44.5 ± 5.1
Al1	53.7 ± 8.3	43.4 ± 8.1	62.3 ± 5.3	46.1 ± 6.5
Al2	61.0 ± 7.1	48.3 ± 6.4	67.7 ± 5.3	50.2 ± 5.7
Al3	62.3 ± 8.3	49.8 ± 8.4	69.5 ± 4.8	51.9 ± 6.9
Al4	61.9 ± 6.8	47.5 ± 5.3	68.3 ± 4.0	49.4 ± 4.6
Av.	59.0 ± 8.5	46.4 ± 7.8	66.4 ± 6.0*	48.7 ± 6.6

*P < 0.05, **P < 0.01.

**Table 5:** Weight-normalized work-per-meter (J*·*(kg*·*m)^−1^).

WC	Smooth floor	Textured mat
Ti	0.155 ± 0.020	0.188 ± 0.020
C	0.162 ± 0.024	0.196 ± 0.025
Al1	0.156 ± 0.023	0.193 ± 0.021
Al2	0.158 ± 0.049	0.219 ± 0.024
Al3	0.155 ± 0.014	0.196 ± 0.019
Al4	0.153 ± 0.027	0.196 ± 0.028
Av.	** 0.157 ± 0.003 **	** 0.198 ± 0.011 **

## Appendices

These appendices put into perspective some aspects of ISO 2631-1:1997 applied to the wheelchair propulsion. It must be emphasized that the norm was developed for the able-bodied population, and its application to wheelchair propulsion is still debated.

### A. Frequency Weighting

The recommended frequency weighting is originally based on discomfort contours obtained from persons subjected to accelerations in different axes and different frequencies [31]. This subjective method based on comfort may seem a serious issue for its application to the SCI population. However, the resulting frequencies are coherent with the resonance frequencies of the seated human body [27, 32]. Therefore, literature on wheelchair vibration tends to agree with this frequency weighting [9, 10, 15, 24]. Some authors prefer to completely avoid it [21].

### B. Axis Selection and Weighting

As introduced in Section 2.3, literature does not agree on the axis to use to assess whole-body vibration when propelling a wheelchair. Wolf et al. [10] considered the vertical (*z*) axis only, Garcia-Mendez et al. [9] considered the total (*x*-*z*) vibration, and Cooper et al. [23] considered the total (*x*-*y*-*z*) vibration. A direct application of ISO 2631-1:1997 gives reason to both first and second authors since no quantitative measure is available to tell if two axes are comparable or not. The standard advises using the total (*x*-*y*-*z*) vibration only to assess comfort.

Having said that, there is a fundamental difference between *x* and *z* vibration in wheelchair propulsion: the latter is induced to the user by ground irregularities, whereas the former is mostly due to the propulsion motion and is voluntarily induced by the user [30].  Figure 3(b) offers a good visual representation of these vibration disparities between both axes. As ISO 2631-1:1997 is based on passive vibration and not on user induced vibration, we believe that assessing the vibration only the vertical (*z*) axis is more advisable than combining *z* with other axes.

### C. Health Considerations

Appendix B of ISO 2631-1:1997 indicates vibration thresholds that quantify the risk of developing health issues due to whole-body vibrations. This can be a starting point to evaluate the maximal allowable vibration for wheelchair propulsion [9], but no validation of the health risks to the SCI population is available at the moment. Therefore, in our work, we chose to avoid comparing the vibration exposure of our subjects to these thresholds and used the standard only to compare wheelchairs among them.
